# Microbes with plastic-degrading and pathogenic potentials are present on plastics in the final polishing pond of a wastewater treatment plant

**DOI:** 10.1186/s40793-025-00737-y

**Published:** 2025-07-01

**Authors:** Jessica A. Wallbank, Fraser Doake, Lloyd Donaldson, Joanne M. Kingsbury, Hayden Masterton, Olga Pantos, Dawn A. Smith, Beatrix Theobald, Louise Weaver, Gavin Lear

**Affiliations:** 1 School of Biological Sciences, University of Auckland, 3a Symonds Street, Auckland, 1010 New Zealand; 2 Institute of Environmental Science and Research, 27 Creyke Rd, Ilam, Christchurch, 8041 New Zealand; 3 Scion, Te Papa Tipu Innovation Park, 49 Sala Street, Rotorua, 3010 New Zealand

**Keywords:** Pathogen rafting, Wastewater treatment plant, Plastisphere, Microbial community

## Abstract

We characterised plastisphere microbial communities in the polishing pond of a municipal wastewater treatment plant, applying prokaryotic 16S rRNA gene, eukaryotic 18S rRNA gene and fungal ITS2 region sequencing to identify changes in microbial biofilm community compositions over time. Pondwater and biofilm from linear low-density polyethylene (LLDPE), nylon-6 (PA), polyethylene terephthalate (PET), polylactic acid (PLA), oxo-degradable linear low-density polyethylene (OXO) and glass were sampled after 2, 6, 26 and 52 weeks of constant immersion. Microbial communities in ambient pondwater differed significantly from those forming biofilms on solid substrates. Biofilm age and depth in the water influenced microbial community compositions. However, no substrate-specific microbial communities were found among glass and plastic polymer types, regardless of artificial ageing. All substrates housed taxa associated with microbes previously reported to biodegrade plastics, being most abundant at two and 52 weeks for bacteria and fungi, respectively. Potential pathogens were found on all substrates, also being most abundant at two and 52 weeks for bacteria and eukaryotes, respectively. Our study highlights that the volume of plastics, more than its polymer form, may be most important when considering plastic's potential impacts on terrestrial and aquatic ecosystems, and for public health.

**Supplementary Information:**

The online version contains supplementary material available at 10.1186/s40793-025-00737-y.

## Introduction

Plastic particles originating from industrial and municipal waste are carried through wastewater treatment plants (WWTPs) before being discharged into rivers and oceans or applied to land as treated waste effluent [110]. Several studies have found plastics in abundance within wastewater effluent following primary and secondary treatments designed to encourage biological settlement and nutrient removal from wastewater sludge [70, 85, 110, 131]. For example, Ruffell et al. [110] found a decrease in plastic abundance from influent to effluent water at three WWTPs in Te Wāipounamu, the South Island (TW-SI) of Aotearoa-New Zealand (A-NZ). However, plastics with an average particle size of 796 ± 64 μm (mean ± stdev) remained prevalent in the effluent water (at an average of 1.3 ± 0.6 particles/L). Whilst Dris et al. [31] concentrations of plastic in the Seine-Centre WWTP in Paris, France, decreased between 83 to 95% from the raw wastewater to the treated effluent, concentrations of plastic in the effluent nevertheless remained high, at 14–50 particles/L. Thus, although it is clear that large amounts of plastics are removed during wastewater treatment, plastic fragments are often moved into the biosolid treatment process, where there is still a risk of plastics entering the terrestrial or aquatic environment [91, 110]. Therefore, current procedures are insufficient to prevent the large-scale release of plastic particles into receiving waterbodies.

Plastics enter wastewater treatment systems from various sources as primary plastic particles, such as beads in cosmetics or synthetic textile fibres in clothing, or as secondary plastic particles from the disintegration or fragmentation of macroplastic via processes such as hydrolysis, abrasion (including mechanical breakage), ultraviolet (UV) photodegradation and biodegradation [67]. Whilst present in the WWTP, plastics provide additional surfaces for colonisation, facilitating the proliferation of opportunistic biofilm colonisers that may have a poorer ability to survive within the water column [56, 61]. Releasing these plastics from wastewater into waterways exposes receiving communities to microbes within the plastisphere. Pathogenic and antibiotic-resistant microbes reside within such ‘plastisphere’ communities, where they may use the plastic as a raft for biofilm formation, hence enhancing their survival and dispersal [61, 66, 145, 151]. A recent study detected a decreased abundance of potential pathogens, such as Campylobacteraceae, in communities associated with plastic particles in effluent as compared to influent samples, whilst other potential pathogens, such as *Acinetobacter*, were similarly abundant in influent and effluent communities but were less abundant on sludge-associated plastics [55]. The spread of antibiotic resistance to pathogens within the plastisphere and subsequently into the environment has been previously reported in freshwater [5, 32], highlighting the need to identify and characterise the microbes entering these environments, along with the potential risks they pose.

Along with the perceived negative impacts of plastisphere communities, plastic provides a persistent platform for biofilm formation, potentially allowing pioneer microbes to utilise carbon available on the plastic surface that either originates from the plastic or binds to the plastic from the surrounding environment [143]. Over 750 prokaryotic and fungal species from such communities have been reported as potential plastic degraders [40]; however, evidence for the degradation of many high molecular-weight plastic polymers remains limited [69]. Additionally, there remains significant potential for microbial diversity to be impacted by nutrient and contaminant accumulation on aquatic plastic surfaces. For example, in a year-long study, Tagg et al. [122] identified *Devosia* sp., an alphaproteobacterium, as being more abundant on plastics than silica control substrates. However, most studies have examined the abundances of prokaryotic or fungal taxa in isolation (but see Wang et al. [135], Guliyev et al. [43]), overlooking the potential variation in community interactions due to temporal shifts and differing plastic types, or interactions with other organisms in the plastisphere. To address this, we explored plastic's potential impact on taxa from both prokaryotic and eukaryotic domains of life. Further, interactions occurring within organismal domains can significantly impact microbial functions and processes, including pathogenicity [117] or protection from pathogens [50], and abilities to degrade complex substrates, including plastics [84]. To this end, we explored the changing community network interactions on different plastic substrates over time.

Our study is the first to characterise plastisphere communities associated with a wastewater treatment plant in Aotearoa-New Zealand (A-NZ). To assess the relative abundances of plastic-specific prokaryotes, fungi and other eukaryotes, we deployed a structure containing five plastic types, along with an inert glass control, into the water of an effluent polishing pond at the Christchurch Wastewater Treatment Plant for one year (sampling from two to 52 weeks). The five polymers used for our study, selected based on their prevalence in municipal and industrial wastewater [110], were linear low-density polyethylene (LLDPE), nylon-6 (a polyamide; abbreviated in our study as PA), polyethylene terephthalate (PET), polylactic acid (PLA) and oxo-linear low-density polyethylene (OXO). Artificially UV-aged plastics were included alongside virgin polymers to determine the impact of plastic deterioration due to environmental exposure on microbial communities. Additionally, we sought to identify the presence, or altered relative abundances of potentially pathogenic, antibiotic-resistant, or plastic-degrading microbes on plastics compared to pondwater communities.

## Materials and methods

### Sampling site and collection

**Fig. 1:**
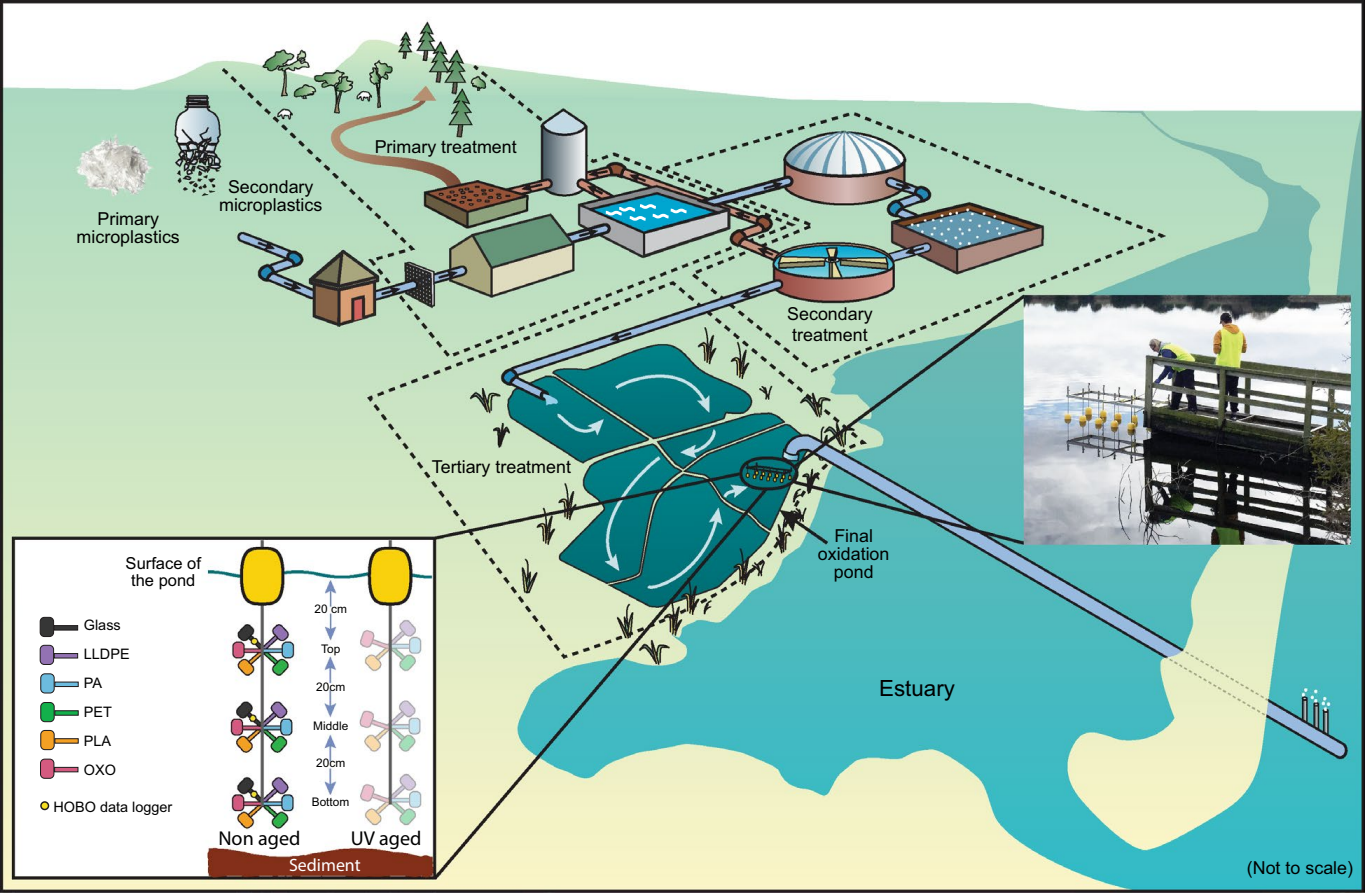
Schematic of the sampling structure, located within a tertiary treatment pond upstream of the wastewater outlet in the ocean. Inset are images showing the deployment of five plastic types in the water column, including both UV-aged and non-aged plastics, and glass control samples. The image is adapted with permission from www.ccc.govt.nz

The location of the deployment was from a jetty in the final polishing pond of a wastewater treatment plant in Ōtautahi-Christchurch, Aotearoa-New Zealand (A-NZ), at a latitude of 43°31′55.2"S and longitude of 172°43′18.8"E. The site is the largest WWTP in Te Wāipounamu, the South Island of A-NZ, with no public access to the deployment location. The plant serves an estimated population of 394,700 (June 2020) and receives approximately 172,000 m^3^ of influent wastewater daily [22]. The wastewater is subjected to primary and secondary treatment, and then travels through five finishing ponds before entering the final maturation pond containing our deployment structure (Fig. 1).

The polymers linear low-density polyethylene (LLDPE), nylon-6 (PA) and polyethylene terephthalate (PET) were selected for use in this study due to their high usage and abundance in the environment [34, 35]; oxo-biodegradable LLDPE (OXO), and polylactic acid (PLA) were included based on their greater biodegradation potential. OXO contains catalytic manganese stearate to increase LLDPE oxidation; all other plastics contained predetermined additives typically included in these products for UV/light stability and degradation prevention (Table S1). Any other contaminants associated with these plastics were at very low levels, i.e., inorganic contaminants were below the limits of detection of thermogravimetric analysis (± 0.5% of the original mass) [68].

While microplastic-sized particles dominate the plastic waste within WWTP effluent, it remains challenging to incubate sufficient microplastic particles under natural environmental conditions to obtain sufficient microbial biomass for experimentation. Thus, we incubated microplastic paddles, instead, as experimental proxy materials. As previously described by Wallbank et al. [134], Bridson et al. [15], Laroche et al. [68] and Theobald et al. [128], all plastics were injection-moulded into rectangular paddles (75 mm by 50 mm by 3 mm) with an integrated arm. Artificial ageing was performed using a Q-Lab Corporation (Ohio) spray accelerated weathering (QUV) tester (ASTM D4329-13/ASTM G154; [8]); this required repeated cycles of 8 h of UVA-340 (preferred irradiance at 340 nm was 0.89 W/m^2^) ultraviolet light at 50 °C (reduced from the usual 60 °C due to the low glass transition temperature of PLA), followed by four hours of condensation (to mimic dew) at 50 °C. Paddle holders were moved across the QUV accelerated weathering tester once per week to minimise variation in exposure at the edges. Paddles were turned within the holders after 400 h to expose the opposite side for a further 400 h. During the experiment, plastic paddles and glass slides (76 mm × 51 mm × 1 mm; low-iron soda-lime float glass; ProSciTech, Australia) were distributed and oriented on stainless-steel poles as described in Wallbank et al. [134], with non-aged and aged plastics being placed on separate poles at depths of 20, 40 and 60 cm. Data loggers (HOBO Pendant MX2202; Onset Computer Corp, Bourne, MA, USA) were attached to the structure at each depth to record light intensity and temperature within the water column at 15-min intervals throughout the experiment. Since the data logger surfaces were cleaned only during sample collection, averages of temperature and illuminance were obtained per depth for the three days following sampling, except for the final timepoint, where averages from three days prior were used due to the removal of the entire sampling structure (Table 1). The structure was deployed on 27 July 2020 (austral winter) and paddles were sampled on four occasions (weeks 2, 6, 26 and 52). A total of 33 biofilm samples were collected per date, or 132 biofilm samples overall (five plastics [LDPE, PA, PET, OXO, PLA] x two conditions [aged, non-aged] x triplicate plastic paddles + three glass slide samples). Three 2 L pondwater samples were also collected into sterile glass bottles at each sampling time.

**Table 1:** Averaged temperature (°C) and light intensity (lux) in the final polishing pond of the Christchurch Wastewater Treatment Plant, three days post-sampling. Datalogger positions Top, Middle and Bottom correspond to the depth from the water surface (20, 40 and 60 cm, respectively)

Time	Datalogger position	Temperature (°C)	Lux	Average minutes of light per day
Deployment	Top	8.85	3835	635
	Middle	8.64	1641	620
	Bottom	8.74	1183	625
2 weeks	Top	10.39	3315	675
	Middle	10.15	1680	665
	Bottom	10.22	656	655
6 weeks	Top	12.18	3126	745
	Middle	11.91	1124	740
	Bottom	11.99	488	730
26 weeks	Top	19.17	4590	915
	Middle	18.78	1205	910
	Bottom	18.77	314	895
52 weeks*	Top	9.54	4381	630
	Middle	9.42	1274	630
	Bottom	9.53	517	615

*Measurements were taken for three days before sampling due to the structure being fully removed after the last sampling time

### Sample processing

As previously described, biomass was extracted from paddles within three hours of collection using flat-edged razor blades, followed by sonication to dislodge any remaining biomass [134]. Samples originating from the glass and plastic paddles were centrifuged at 4,500 × *g* for 10 min at 4 °C, the supernatant was discarded, and the pellets were stored at − 80 °C until required. Pondwater was filtered (approximately 300 ml per filter) through 0.2 µm filter membranes (Supor® 200, Whatman) using a vacuum pump. Individual membranes were placed into sterile 5 ml centrifuge tubes and also stored at − 80 °C until required.

### DNA extraction

Microbial DNA was manually extracted from up to 250 mg biomass using DNeasy PowerSoil Pro kits (Qiagen, Hilden, Germany), according to the manufacturer’s instructions, apart from the mechanical lysis step, which was performed using a TissueLyser II (Cat No. 85300; Qiagen, Hilden, Germany) for 2 min at 30 Hz. When sample biomass was < 100 mg, 100 μl UltraPure DNase/RNase-Free distilled water (Cat No. 10977015; Invitrogen, Thermo Fisher Scientific, Waltham, MA, USA) was used to resuspend the pellet and the entire volume was used for extraction. Filters used to process water samples were placed into the bead tubes directly using sterile tweezers. DNA was extracted from 143 samples (i.e., 33 biofilm samples, plus three pondwater samples for the four sampling times), alongside sampling blanks and DNA extraction kit control blanks, following the manufacturers’ instructions, each eluted in 100 μl elution buffer. One glass sample was missing from the structure at 12 months; therefore, only two glass replicates were sampled at this time. DNA samples were aliquoted and stored at −80 °C until required.

### PCR and DNA sequencing

Polymerase chain reactions (PCRs) were performed to amplify: (i) the hypervariable V4 region of the prokaryotic small-subunit of ribosomal RNA (16S) genes, using the universal amplicon primer pair: 515 F and 806RB (Table S2), (ii) the fungal internal transcribed spacer 2 (ITS2) region of the nuclear ribosomal gene using the primer pair: fITS7 and ITS4 (Table S2), and (iii) total hypervariable V4 region of the eukaryotic small-subunit of ribosomal RNA (18S) genes, using the primer pair: Uni18SF and Uni18SR (Table S2). All primers were modified to contain the Illumina adapter sequences required for amplicon sequencing (Kozich et al., 2013). All PCRs, including controls, were conducted in total reaction volumes of 25 µl containing 0.25 µl MTP™ Taq DNA Polymerase, 2.5 µl 10X MTP™ Taq buffer (Invitrogen, Thermo Fisher Scientific, Waltham, MA, USA), 0.5 µl dNTPs (10 mM, Sigma-Aldrich New Zealand Co, affiliate of Merck KGaA, Darmstadt, Germany), 0.75 µl of each primer (10 µM), 1 µl template DNA and up to 25 µl of UltraPure DNase/RNase-Free distilled water, under the thermocycling conditions corresponding to their target amplicon. To verify amplicon sizes and check for contamination, 4 μl of PCR products were run on a 0.8% (w/v) agarose gel, stained using SYBR SAFE DNA Gel Stain (Invitrogen, Thermo Fisher Scientific, Waltham, MA, USA) and visualised under UV light using a GelDoc imaging system. Samples were purified using DNA Clean and Concentrator-5 kits (Zymo Research, Irvine, CA, USA) and PCR products eluted in 15 μl DNA elution buffer, according to the manufacturer’s instructions. Samples were then diluted to 3 ng/µl using UltraPure DNase/RNase-Free distilled water, alongside a negative control of UltraPure DNase/RNase-Free distilled water for the multiplexing and sequencing steps in each library. Mock microbial standards (ZymoBIOMICS Microbial Community and DNA standards) were included as controls to identify bias in DNA extraction, amplification and sequence runs, respectively (Zymo Research, Irvine, CA, USA). Each target amplicon was amplified from the mock standards, as previously mentioned, and included on each relevant run. DNA sequencing was conducted by Auckland Genomics Facility (The University of Auckland, New Zealand) on an Illumina MiSeq instrument using 2‐by‐300‐bp V3 chemistry. A unique combination of Nextera XT dual indices (Illumina Inc., San Diego, CA, USA) was attached to the DNA of each sample before DNA sequencing to allow sample multiplexing by the sequencing provider, who subsequently provided us with demultiplexed raw sequence reads.

### Accession numbers

Raw sequence reads are available from the NCBI Sequence Read Archive (SRA) under the BioProject ID PRJNA1161030.

### Processing and analysis of DNA sequence data

Trimmomatic (version 0.39; [12]) was used to remove sequence adaptors using the parameters: ILLUMINACLIP:2:40:15. The ‘DADA2’ package [18] in R (version 4.2.1; [108]) was used to trim prokaryotic and eukaryotic primers and process reads, following the package instructions. Fungal primers were removed using Cutadapt [77] before being processed using DADA2. Sequence read quality was inspected, and reads were filtered, allowing a maximum estimated error (“maxEE”) of 2 per 100 bp. To estimate sequence error rates, samples were pooled to achieve convergence of the parametric error model. All samples were dereplicated and pooled together for amplicon sequence variant (ASV) inference to reduce low sampling bias. Paired-end reads were subsequently merged, chimeric sequences removed and an ASV table constructed. ASVs were defined as clusters with 100% DNA sequence identity. Taxonomic assignment was performed against the SILVA reference database for prokaryotic 16S rRNA sequence reads (version 138.1; [82]) and eukaryotic 18S rRNA sequence reads (version 132; [89]), and the UNITE reference database was used for fungal ITS sequence reads (general FASTA release version 9.0 (18.07.2023); [1]), using the native implementation of the naïve Bayesian classifier method.

### Bioinformatics and statistical analyses

#### Data preparation

All ASV tables were filtered only to include the kingdoms relevant to each target amplicon: bacteria and archaea (for 16S rRNA gene sequences), fungi (for ITS2 region sequences) and eukaryotes (for 18S rRNA gene sequences). All ASVs unclassified to the kingdom level and those assigned to the family level as mitochondria, or order as chloroplast, were removed. The “isContaminant” function of the ‘decontam’ package (version 1.22.0; [27]) was used to identify and remove contaminant sequences found in PCR water sequencing controls, PCR negative assays, extraction kit controls and field sampling blanks that were not removed during previous filtering. Statistical analyses and visualisation were achieved using R (version 4.3.2; [108]). For alpha diversity analyses only, ASV tables were rarefied to 1000, 2945 and 250 for prokaryotes, fungi, and eukaryotes, respectively. To accommodate differences in sequencing depth and ensure comparable diversity, cumulative sum scaling (CSS) normalisation was performed on the non-rarefied data using the ‘metagenomeSeq’ R package (version 1.43.0; [102]).

### Quantitative analyses

Alpha diversity analyses were performed to investigate taxonomic richness and evenness within and among samples; Chao1, Shannon and inverse Simpson statistics were calculated using the “estimate_richness” function of the ‘phyloseq’ package (version 1.42.0; [83]).

For analyses of beta diversity, Bray–Curtis dissimilarity matrices of CSS-transformed data were constructed and permutational multivariate analysis of variance (PERMANOVA) with 999 permutations was performed using the “adonis2” function in the ‘vegan’ R package (version 2.6.4; [99]); data were visualised on an NMDS plot using the ‘ggplot2’ package (version 3.4.3; [139]). Pairwise PERMANOVAs were performed using the ‘vegan’ wrapper “pairwise.adonis” function of ‘pairwiseAdonis’ (version 0.4.1; [78]) to determine statistical significance (*P* < 0.05) between microbial community compositions due to different substrates, biofilm age, plastic condition, and paddle depths. To determine which variables had the most influence on the prokaryotic, fungal and eukaryotic communities, an average centroid of each variable was used to calculate Bray–Curtis dissimilarity. To better ascertain any effects related to the ‘Age’, ‘Condition’ and ‘Depth’ of the different plastic polymers, glass and pondwater sample data were then excluded from the averaged centroids (since UV-ageing assays were not performed on glass or water samples). To evaluate the significance of these factors for microbial community composition, Bray–Curtis dissimilarity matrices were determined for all samples and PERMANOVAs were completed. The homogeneity of multivariate variance was assessed using the vegan ‘betadisper’ function.

To determine the presence of any microbes closely related to previously reported plastic-degraders within our samples, taxonomy tables were searched against a database of putative plastic-degrading organisms (PlasticDB; [40]); potential bacterial and fungal plastic-degraders were identified at genus and species levels. To determine the presence of potentially pathogenic microbes within our samples, ASVs were screened at the genus level against the World Health Organisation (WHO) global pathogens priority list for antibiotic-resistant bacteria [141], the WHO fungal pathogens priority list [142] and a local notifiable diseases list [75].

To detect significant microbial associations (e.g., co-occurrence patterns), ASV relative abundances were averaged by substrate (i.e. glass, plastic and pondwater) at each timepoint To remove less abundant taxa that may produce spurious interactions, ASVs with < 1% relative abundance within each network were removed and differences in the co-occurrence patterns of remaining ASVs were tested, following the approach of Williams et al. [140]. Spearman’s correlation coefficients were generated to represent the strength of co-occurrences between all ASV pairs using the R package ‘stat’ (version 4.4.1 [108]). These co-occurrences were only deemed robust if statistically significant (*P* < 0.01) and where the Spearman’s correlation coefficient (rho or ρ) was greater than 0.7. Spearman’s correlation coefficient distance matrices were then calculated using the “spearman.dist” function of the ‘bioDist’ R package (version 1.78.0; [30]); PERMANOVAs and pairwise PERMANOVAs were performed as already described to investigate significant differences in microbial co-occurrence between substrate types. Whilst this method may produce some spurious interactions [38], pairwise interactions were used to retain only those that were consistent across the sample types. Datasets were converted into the network format, correlation values were added and network statistics were computed using the R package ‘igraph’ (version 2.0.3; [25]). The total number of nodes and edges were calculated to show the number of ASVs and co-occurrences within each network, and to determine whether network complexity varies based on substrate. To further investigate each network, ‘average degree’ was determined to quantify correlations among ASVs by calculating the strength of the edges of each node compared to the number of connected nodes. To determine whether observed networks represent actual co-occurrences, the ‘erdos.renyi.game” function of ‘igraph’ was performed to generate one thousand random networks comparable in size to our networks using an Erdös-Rényi model, as per Lupatini et al. [72]. Networks were visualised in the Fruchterman-Reingold layout using the interactive software Gephi (version 0.10.1; [9]).

## Results

### Taxonomic assignment and relative abundance of bacterial, fungal and eukaryotic reads

All eight bacterial genera were identified within our mock samples, with *Lactobacillus* assigned as *Limosilactobacillus*. The bacterial mock community DNA standard showed comparable relative abundances as expected, with a slightly higher relative abundance of *Limosilactobacillus* (Figure S1). The mock extraction highlighted lower relative abundances of *Enterococcus* and *Listeria*, supporting previous indications of bias against Gram-positive bacteria due to their greater cell wall thickness [44]. The fungal mock community DNA standard contained only reads assigned to *Cryptococcus*, not identifying *Saccharomyces*, highlighting potential primer bias and mismatch against Ascomycota, as previously suggested by Ihrmark et al. [49] and Taylor et al. [126]. Analysis of the mock community DNA amplified using the eukaryote primers generated no reads that could be identified past the kingdom level. Thus, no reads were retained after DADA2 processing for fungal and eukaryotic mock extractions.

DNA sequences originating from negative control samples (assays of sampling blanks, DNA extraction kit blanks, PCR negative controls and water sequencing blanks) that were not filtered out during DADA2 pre-processing were assessed for their potential contaminants. No bacterial or fungal ASVs were identified as contaminants. A total of 4488 bacterial ASVs were identified, and 960 ASVs were filtered out as chloroplasts, leaving 3528 true bacterial ASVs within the samples. After quality filtering, 1927 and 1557 ASVs were obtained for fungal and eukaryotic analyses, respectively. During rarefaction, 21 bacterial and 22 eukaryotic samples were removed for having inadequate DNA sequence reads; no fungal samples were removed. Rarefaction curves for the bacterial (Figure S2A), fungal (Figure S2C) and eukaryotic (Figure S2E) communities indicated that adequate sequencing depth was achieved for most samples, ensuring that most taxa were being represented.

### Comparison of bacterial, fungal, and eukaryotic community richness, evenness and diversity across substrates and ages

Across all the samples collected, ANOVA indicated significant differences in microbial community richness and evenness comparing substrate types (Table 2); a significant interaction term was detected between substrate type and biofilm age, as pondwater and glass communities became less diverse over time, whilst plastic biofilms appeared to maintain the same level of diversity (Figure S2B, D, E). No significant differences in bacterial community indices were determined by comparing all samples collected at different dates (i.e. the term ‘Age’, Table 2). However, fungal, and eukaryotic diversity increased significantly over time, with samples becoming richer and more even (Table 2; Figure S2B, D, F).

**Table 2:** Analysis of variance (ANOVA) investigating substrate, age, depth and their interaction terms for ‘all samples’, ‘biofilm samples only’ and ‘plastic-associated samples only’, as relating to three community diversity metrics

Terms	Prokaryotes			Fungi			Eukaryotes		
	Chao1	Shannon	Inverse Simpson	Chao1	Shannon	Inverse Simpson	Chao1	Shannon	Inverse Simpson
All samples:									
Age	0.671	0.842	0.959	**0.001**	**0.001**	**0.001**	**0.001**	**0.001**	**0.001**
Substrate	**0.005**	**0.009**	**0.001**	0.236	0.357	**0.036**	**0.001**	**0.002**	**0.001**
Age*Substrate	**0.015**	**0.030**	**0.001**	0.670	**0.001**	**0.004**	**0.001**	**0.001**	**0.001**
Pondwater samples removed:									
Age	0.097	0.092	0.077	**0.001**	**0.001**	**0.001**	**0.001**	**0.001**	**0.001**
Depth	**0.001**	**0.001**	**0.001**	**0.001**	**0.001**	**0.001**	0.074	**0.001**	**0.001**
Substrate	0.883	0.911	0.884	0.058	**0.019**	**0.010**	**0.001**	**0.004**	**0.001**
Age*Depth	**0.039**	**0.006**	**0.018**	**0.001**	**0.001**	**0.001**	0.495	**0.003**	**0.001**
Age*Substrate	0.426	0.412	0.242	**0.036**	0.412	0.062	**0.001**	**0.001**	**0.001**
Depth*Substrate	0.500	0.316	0.378	0.299	0.226	0.404	0.285	0.446	0.208
Age*Depth*Substrate	0.811	0.657	0.290	0.577	0.589	0.121	0.078	0.799	0.467
Pondwater and glass samples removed:									
Age	**0.029**	**0.022**	**0.015**	**0.001**	**0.001**	**0.001**	**0.001**	**0.001**	**0.001**
Depth	**0.001**	**0.001**	**0.001**	**0.001**	**0.001**	**0.001**	0.143	**0.001**	**0.001**
Substrate	0.881	0.920	0.789	0.181	**0.020**	**0.014**	**0.001**	**0.002**	**0.001**
Age*Depth	0.096	**0.015**	0.061	**0.001**	**0.001**	**0.001**	0.487	**0.011**	**0.001**
Age*Substrate	0.779	0.846	0.624	0.630	0.336	0.097	**0.001**	**0.001**	**0.001**
Depth*Substrate	0.477	0.326	0.366	0.357	0.195	0.394	0.227	0.435	0.316
Age*Depth*Substrate	0.784	0.591	0.240	0.559	0.592	0.385	0.053	0.785	0.511

Significant *P* values (*P* < 0.05) are in bold

Pondwater sequence data were removed to compare communities forming a biofilm on solid substrates (i.e., on plastic or glass), which revealed significant differences in fungal and eukaryotic community richness and evenness relating to substrate type, biofilm age and their interaction (Table 2). Sample depth was significant in driving differences in microbial richness and evenness, with significant interaction terms identified between biofilm age and depth. When glass-associated community data were also removed, results suggested that biofilm age, depth, and their interaction term, were most significant in influencing microbial community taxonomic richness and evenness. ANOVA also revealed that plastic substrate affects the fungal and eukaryotic community diversity but not bacterial. A significant interaction term was identified between biofilm age and substrate type driving the evenness of eukaryotic communities, with increased diversity within communities present in pondwater and on LLDPE, PA, OXO, and glass at 52 weeks. Overall, substrate type, biofilm age, and depth were important factors driving microbial communities'species richness and evenness on plastics in the final polishing pond of the wastewater treatment plant.

### Bacterial, fungal and eukaryotic community variation across substrate, ages and depth

**Fig. 2:**
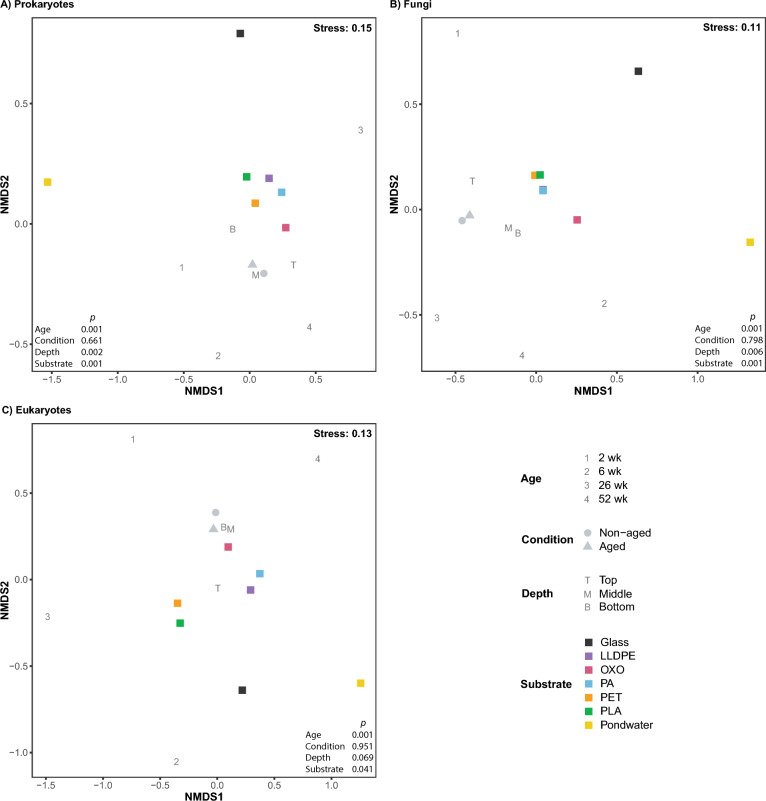
Variations in **A** prokaryotic, **B** fungal, and **C** eukaryotic communities in the final polishing pond of the Christchurch Wastewater Treatment Plant in Ōtautahi-Christchurch, Aotearoa-New Zealand (A-NZ). Plots are derived from non-metric multidimensional scaling of ASV data using Bray–Curtis distances. Data are averages representing one point per variable (i.e. ‘T’ represents an average of data from all samples collected at the shallowest depth)

**Fig. 3:**
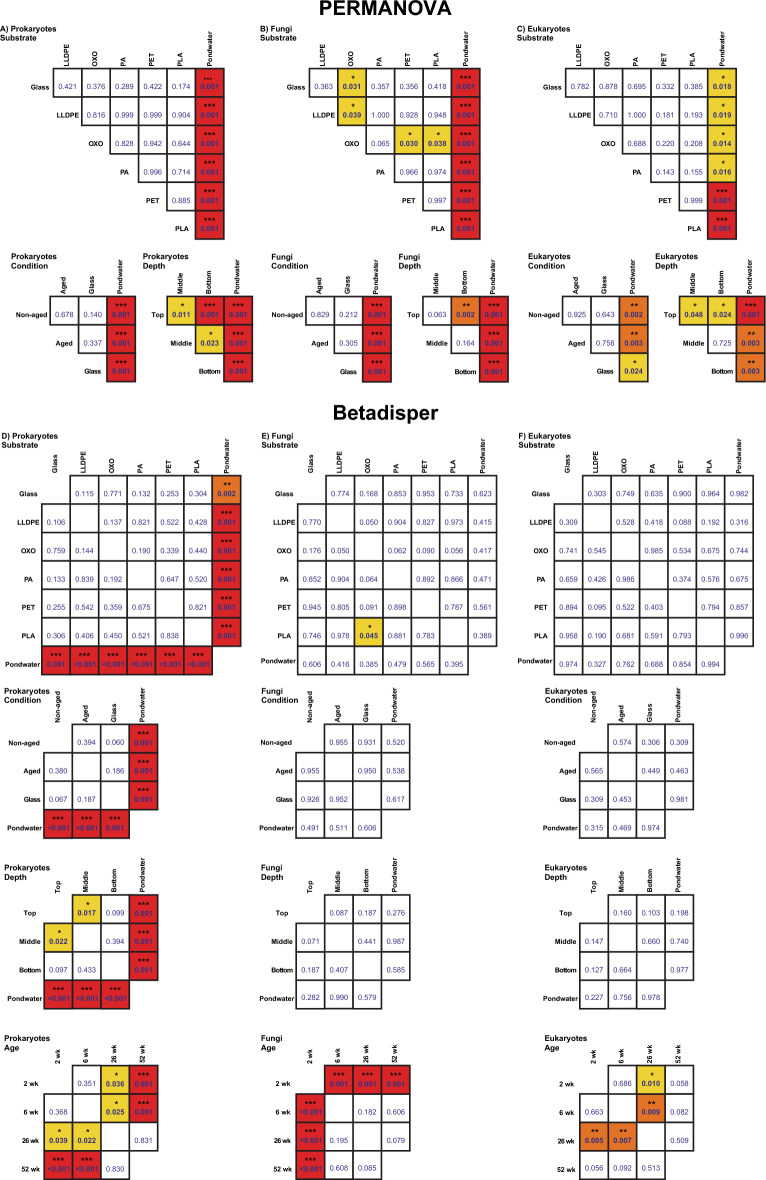
Pairwise PERMANOVA comparisons and betadisper analyses on Bray–Curtis dissimilarity matrices. Colour represents significance: red – *P* < 0.001 (***), orange – *P* < 0.01 (**), yellow – *P* < 0.05 (*), with observed betadisper p-values below diagonal ‘lines’ of blank boxes and permuted betadisper p-values above

Non-metric multidimensional scaling (NMDS) analysis showed pondwater data clustering separately from microbial biofilm community data (PERMANOVA; bacteria and fungi, *P* = 0.001; eukaryotes, *P* = 0.041; Fig. 2A, B and C). This is further supported by pairwise PERMANOVA (bacteria and fungi all *P* ≤ 0.001 and eukaryotes all *P* ≤ 0.02; Fig. 3) for pondwater vs all other substrates separately. Biofilm age was also statistically significant in shaping the microbial communities at all times, regardless of whether pondwater and glass sample data were removed or not (PERMANOVA *P* = 0.001 for bacteria, fungi and eukaryotes; pairwise PERMANOVA *P* = 0.001 for all comparisons; Fig. 2A, B, C, and S3). Similar findings were reported for betadisper analysis (Fig. 3), suggesting that PERMANOVA results may be attributed to variations in the dispersion of the data.

Microbial communities did not appear to be influenced by whether the plastic was artificially UV-aged (i.e. its condition before entering the polishing pond (Fig. 2), as reflected by no significance observed by PERMANOVA or beta dispersion tests (Fig. 3, bacteria *P* ≥ 0.06; fungi *P* ≥ 0.212; eukaryotes *P* ≥ 0.306). Microbial communities in pondwater were significantly different to those on non-UV-aged plastics, UV-aged plastics and glass, as shown by pairwise PERMANOVA, with significance also observed in the dispersion of bacterial communities on aged plastics, non-aged plastics and glass compared to pondwater. Biofilms on glass, however, were shown to have no significant differences overall compared to microbial communities present on the two plastic conditions (i.e. non-aged and UV-aged plastics).

Due to the mixing of pondwater obviating any depth effect, pondwater triplicate samples were each collected from across the three depths rather than from three individual depths. Hence, pondwater samples were not assigned specific sampling depths; they were simply labelled as ‘Pondwater’. Depth was an important factor in driving the diversity of the biofilm plastisphere bacterial, fungal and eukaryotic communities (PERMANOVA; *P* = 0.001, 0.006 and 0.015 for bacteria, fungi and eukaryotes, respectively), with pairwise comparisons revealing that pondwater was significantly different to biofilm samples collected at the three depths (pairwise PERMANOVAs; Fig. 3). Significance was also observed between microbial communities on top paddles compared to middle and bottom paddles for bacteria and eukaryotes, with significant differences in fungal communities only observed between top and bottom paddles. Bacterial communities on the middle paddles also significantly differed from those on the bottom paddles; however, no significance was observed between those depths for fungi or eukaryotes. Data dispersion among biofilm samples at different paddle depths was not significantly different (bacteria *P* = 0.052; fungi *P* = 0.155; eukaryotes *P* = 0.223); therefore, homogenous dispersion is likely between paddle depths.

**Fig. 4:**
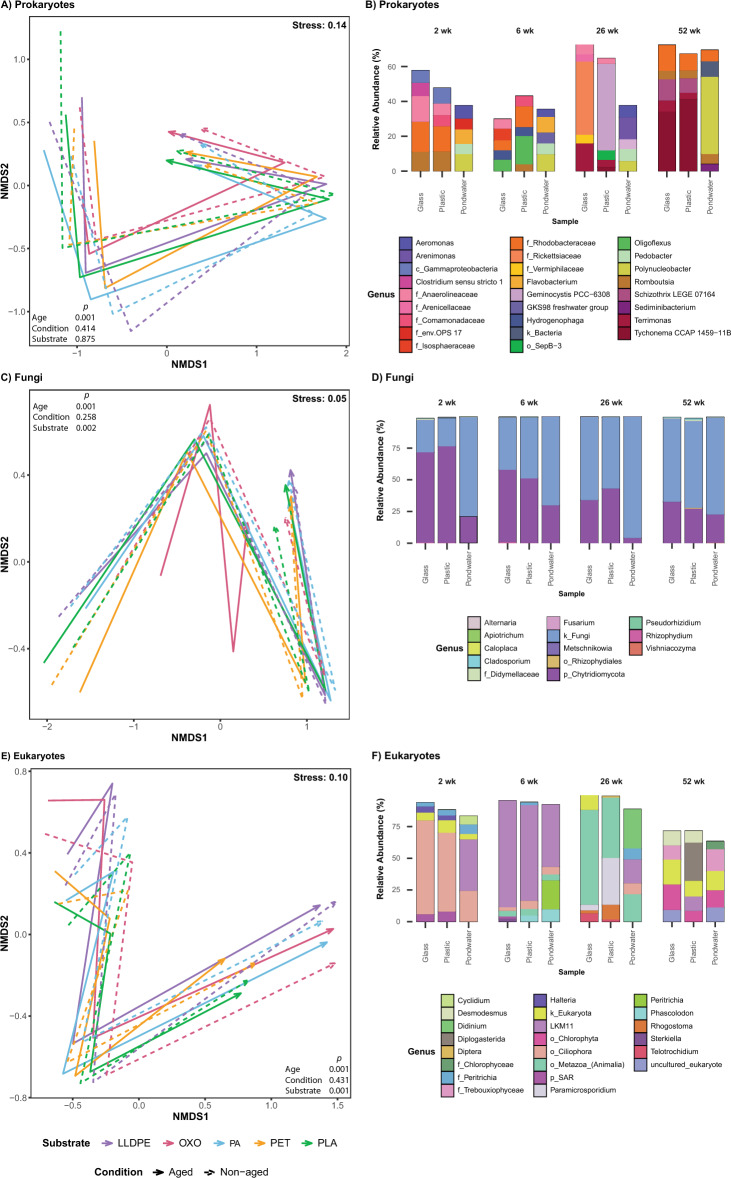
Non-metric multidimensional scaling (NMDS) ordination for the visualisation of Bray–Curtis similarities of **A** prokaryotic, **C** fungal, and **E** eukaryotic communities on substrates in the final polishing pond of the Christchurch Wastewater Treatment Plant in Ōtautahi-Christchurch, Aotearoa-New Zealand (A-NZ). Centroids of each substrate per timepoint were averaged and labelled according to substrate type (colour) and substrate condition (i.e. exposed to UV treatment or not; line type), with trajectories indicating the change in communities over time. Data from different conditions and sampling depths were combined for plastic, glass and pondwater substrates separately to plot average relative abundances (%) of the five most dominant **B** prokaryotic, **D** fungal and **F** eukaryotic genera present per sample at each different biofilm age. Each bar represents the average relative abundance, collected four times over a year. Each colour corresponds to a particular genus

Pondwater data were removed, and analyses were repeated to determine which factors influenced the microbial communities on plastic and glass alone. As the artificial ageing of plastic was determined not to be significant (Figs. 4A, C and E), only substrate type, biofilm age and depth were investigated. Substrate type was not a significant factor in shaping the bacterial communities present in biofilms with glass present (*P* = 0.291) or without (*P* = 0.649); therefore, plastic substrate type appears to have little influence in determining the diversity of bacteria present. Substrate type was determined to be significant for fungal and eukaryotic communities, however (PERMANOVA; *P* = 0.001), along with its interacting term with age (PERMANOVA; *P* = 0.001). No significant difference was determined for data dispersion among biofilm communities due to substrate with (bacteria *P* = 0.451; fungi *P* = 0.238; eukaryotes *P* = 0.802), or without glass (bacteria *P* = 0.541; fungi *P* = 0.168; eukaryotes *P* = 0.695), suggesting significant PERMANOVA results observed are due to differences in ‘data location’ as opposed to ‘data dispersion’. Biofilm age and depth, along with their interaction, were determined to be the most significant variables driving the composition of the bacterial, fungal, and eukaryotic communities (PERMANOVA; *P* = 0.001).

To investigate the effect of depth on microbial communities, the relative abundances of phyla were plotted. Proteobacteria dominated at the earliest time across all depths, with high abundances of Firmicutes, Bacteroidota, and Chloroflexi observed. After six weeks, Firmicutes, Bacteroidota and Chloroflexi were only observed in low abundance on middle and bottom paddles, with an increase of Bdellovibrionota preferentially on top plastic paddles and Planctomycetota on middle and bottom paddles (Figure S4A). Cyanobacteria were the dominant phylum on plastic paddles at 26 weeks and all biofilms at 52 weeks, with higher abundance on plastics than glass. Interestingly, at 26 weeks, Cyanobacteria are less abundant on the shallowest non-aged plastic whilst highly dominant on the aged plastic at the same depth, except for non-aged LLDPE (Figure S4B). The opposite is observed on the bottom paddles. However, at 52 weeks, all top paddles have a high abundance of Cyanobacteria, which was observed to decrease with depth. Of the fungi, Chytridiomycota was the most abundant phylum at two and six weeks, with unidentified fungi becoming more dominant over time, with no distinct differences between depth or plastic conditions (Figure S5A). The eukaryotic clade comprised of Stramenopila, Alveolata and Rhizaria (SAR) was the most dominant at two weeks, after which Opisthokonta dominated (Figure S5B). However, at 52 weeks, Archaeplastida was highly abundant in all pondwater and biofilm samples, apart from PET and PLA (Figure S5B).

To determine which microbes may be driving the significant differences observed between plastic and glass at different biofilm ages compared to pondwater, the relative abundances of the top five most abundant genera within glass, plastic and pondwater communities, on average, were plotted for each sampling time. The most dominant genera on plastic and glass were distinct at each biofilm age (Fig. 4B, D, F). On average, the most abundant taxa in pondwater were distinct from those in biofilm communities: *Aeromonas*, *Arenimonas*, *Flavobacterium*, *Pedobacter*, *Polynucleobacter* and *Sedminibacterium* spp. (Fig. 4B). Bacterial genera belonging to class Gammaproteobacteria, families Anaerolineaceae and Rhodobacteraceae, and genus *Romboutsia* spp., were all most abundant, in relative terms, in both glass and plastic-associated communities at two weeks, suggesting the significant differences we observed in community diversity among these substrate types is primarily driven by rare taxa present within the different communities. *Oligoflexus*, *Hydrogenophaga* and Rhodobacteraceae genera were the most abundant bacteria, in relative terms, in six-week-old communities on glass and plastics. *Terrimonas* spp. and Anaerolineaceae genera were the only taxa in the top five genera present on both glass and plastics at 26 weeks. The most abundant genus in the plastic communities at 26 weeks was *Geminocystis*, which was also abundant in pondwater then. At 52 weeks, the top five bacterial genera were shared between glass and plastics; *Tychonema* spp. was the most abundant in biofilm communities, alongside *Terrimonas*, *Schizothrix*, *Romboutsia* spp. and Rhodobacteraceae genera. More than 60% of the bacterial biofilm communities present comprised the top five genera after 52 weeks, indicating the formation of a generalist community (Fig. 4D). *Cladosporium* spp. and Didymellaceae genera were detected in all biofilm samples, and in pondwater at two and 52 weeks. Most fungal ASVs remained unassigned at the genus level due to a lack of fungal identification, with over 80% of ASVs unassigned past the kingdom Fungi and the phylum Chytridiomycota. At the genus level, over 75% of ASVs comprised the top five most abundant eukaryotic genera at two, six and 26 weeks (Fig. 4F). Eukaryotes not assigned at the phylum level were observed on all substrates at two and 52 weeks and on glass at 26 weeks. *Halteria* spp. were detected on biofilm samples only; *Cyclidium* spp. were detected in pondwater at two weeks. Several taxa, such as those belonging to the phylum SAR, order Ciliophora, and family Peritrichia, were present in all samples at two weeks. Likewise, Ciliophora was abundant at six weeks in all samples, along with the genus *LKM11* and taxa belonging to the order Metazoa. *Sterkiella* spp. were found exclusively on glass samples, whereas *Phascolodon* spp. were detected on plastics and in pondwater; *Peritrichia* spp. were observed in pondwater only, at six weeks. *Paramicrosporidium*, *Rhogostoma* and *Telotrochidium* spp. were most abundant in biofilm communities, whilst *Didinium* spp. were found only in pondwater at 26 weeks. At 52 weeks, *Desmodesmus* spp. were abundant on glass and plastics, whilst *Diplogasterida* and *LKM11* spp. were abundant on plastics only.

### Screening for potential plastic-biodegraders in samples

**Fig. 5:**
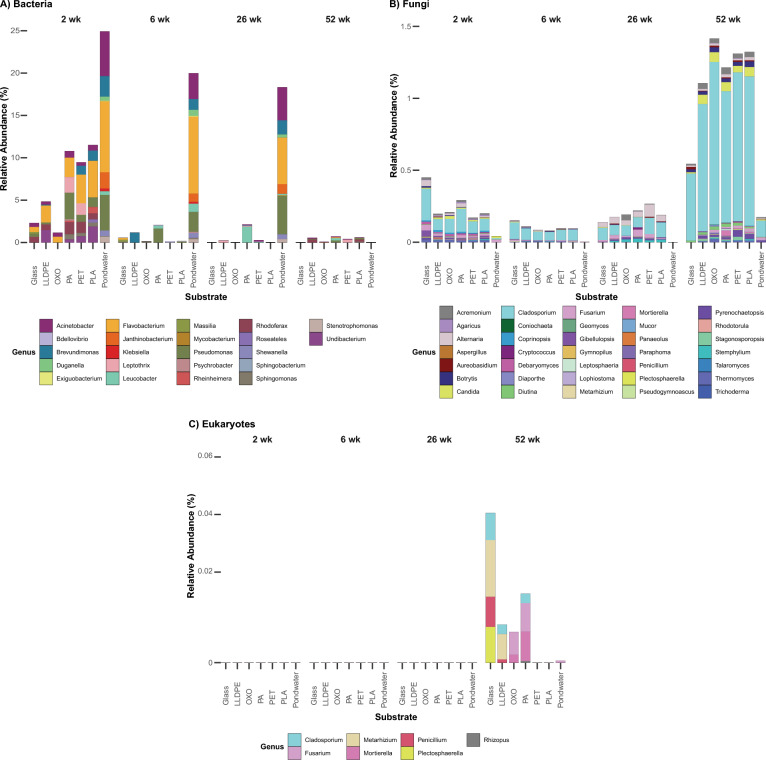
Average relative abundances (%) of previously reported **A** bacterial, **B** fungal, and **C** eukaryotic plastic-biodegrading genera within communities from the final polishing pond of the Christchurch Wastewater Treatment Plant in Ōtautahi-Christchurch, Aotearoa-New Zealand (A-NZ). Each bar represents the stacked relative abundance averaged within each substrate, collected at four different times. Each colour corresponds to a particular genus

Previously reported plastic-degrading microbes were identified within our samples. At the genus level, 22 bacteria (Fig. 5A) and 36 fungi (Fig. 5B and C) were identified. Interestingly, presumed plastic-biodegrading bacteria were most abundant after two weeks, representing nearly 25% of the community in pondwater, far more than the plastic-associated communities (Fig. 5A). Potential plastic-degrading fungi were most abundant after 52 weeks, with a higher abundance in biofilms (Fig. 5B and C). The most common presumptive plastic-degrading bacteria were members of *Acinetobacter*, *Brevundimonas, Flavobacterium* and *Pseudomonas* spp., whereas for fungi, members of *Cladosporium* spp. dominated.

Overall, a higher relative abundance of previously reported plastic-degrading bacteria was present in pondwater relative to glass and plastic, although some appeared to show preferential presence on plastics. The relative abundance of proposed plastic-degrading fungal taxa was greatest at 52 weeks, with some preferential settlement on plastics compared to glass and pondwater; however, they were in far lower abundance than proposed bacterial plastic-degraders.

### Screening for potential pathogens

**Fig. 6:**
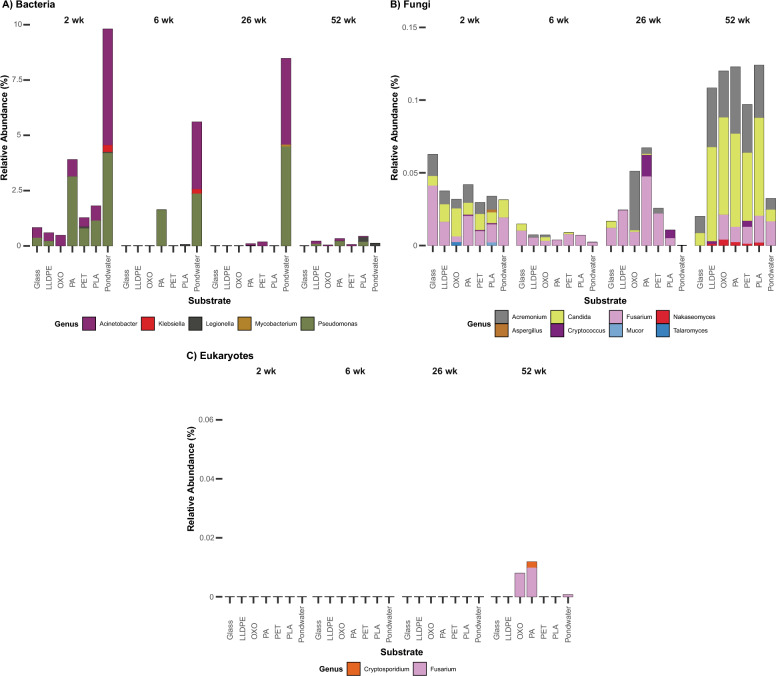
Average relative abundances (%) of potential pathogenic **A** bacterial, **B** fungal, and **C** eukaryotic genera within communities in each sample from the final polishing pond of the Christchurch Wastewater Treatment Plant in Ōtautahi-Christchurch, Aotearoa-New Zealand. Each bar represents the average relative abundance of potential pathogenic taxa associated with each substrate, collected four times over a year. Each colour corresponds to a particular genus

Close relatives of bacterial and fungal pathogens from the WHO priority pathogens and Aotearoa-New Zealand’s notifiable bacteria, fungi and non-fungal eukaryotes lists were identified within our samples at the genus level. Five genera of potential bacterial pathogens were identified, which are more prevalent in relative terms within the pondwater at two, six, and 26 weeks (Fig. 6A). After two weeks of incubation, *Acinetobacter and Pseudomonas spp.* were abundant on most plastics. *Klebsiella* and *Mycobacterium* spp. were only identified in pondwater. However, *Legionella* spp. were more relatively abundant on PLA at 52 weeks. No putative bacterial pathogens were detected on the glass after two weeks. Eight fungal genera associated with fungal pathogenicity were identified, with a higher abundance found on plastics at the final sampling date as the biofilm matured (Fig. 6B). *Acremonium*, *Candida* and *Fusarium* spp. were detected in pondwater and biofilm communities (Fig. 6B). *Aspergillus*, *Cryptococcus*, *Mucor*, *Nakaseomyces*, and *Talaromyces* spp. were found on plastic alone; however, they were in very low relative abundance. Interestingly, *Fusarium* was the only fungal genus identified within the amplified 18S rRNA gene samples (Fig. 6C). *Cryptosporidium* was the only pathogenic non-fungal eukaryote found and was only observed in low abundance on PA at 52 weeks. Overall, a higher abundance of putative bacterial pathogens was present at two weeks on solid substrates and in pondwater. In contrast, potential eukaryotic pathogens, including fungi, were more abundant at 52 weeks on plastics.

### Microbial community complexity within the plastisphere

**Fig. 7:**
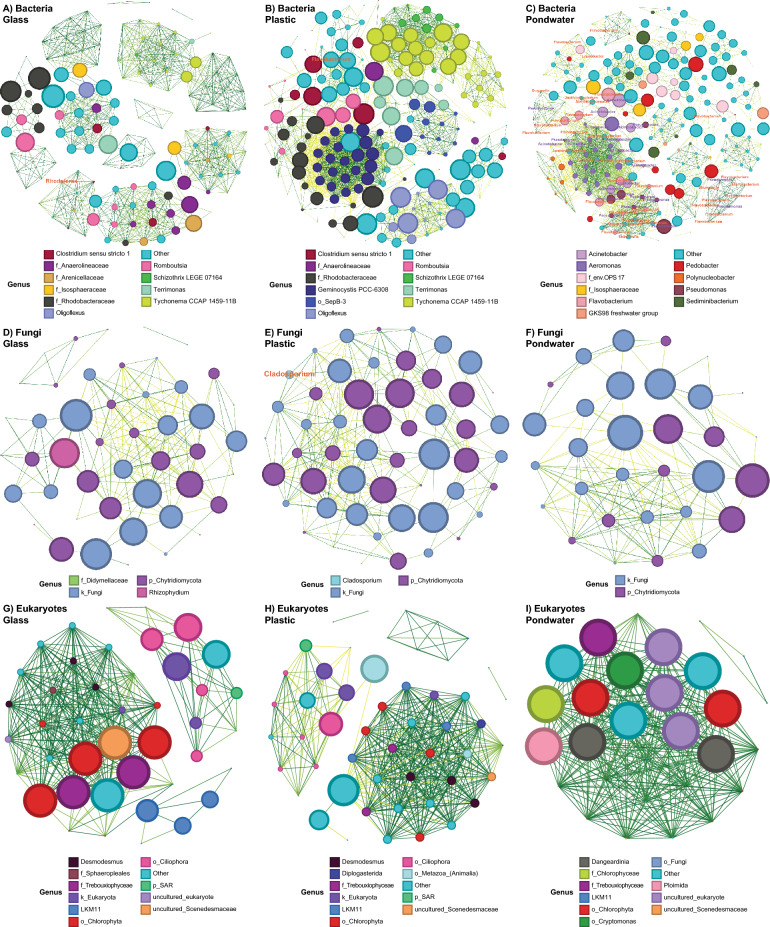
Network interactions of **A**, **B**, **C** bacterial, **D**, **E**, **F** fungal and **G**, **H**, **I** eukaryotic ASVs found across the **A**, **D**, and **G** glass, **B**, **E**, and **H** plastic biofilms and **C**, **F** and **I** pondwater communities. A connection between circles indicates a strong (ρ > 0.7) and significant (*P* < 0.01) Spearman’s correlation. Each circular node represents a core bacterial ASV. The size of the circle is proportional to the betweenness centrality value. Node colours represent the ten most abundant genera, with all other taxa assigned to the ‘other’ colour. Lines connecting two nodes are coloured across a gradient in relation to Spearman’s correlation between ASVs, from negatively correlated (yellow) to positively correlated (green). ASV labels in orange indicate previously reported plastic-biodegrading microbes. Purple ASV labels indicate genera that have been previously reported as plastic-degraders and are also potential pathogens. Network parameters were measured (Figure S6)

Microbial communities present on plastics and in pondwater are more complex than communities on glass (Fig. 7); a higher number of significant nodes (ASVs) and edges (representing significant correlations between ASVs or nodes) were observed in the microbial plastisphere and bacterial pondwater networks compared to glass (Figure S6A and B), suggesting more significant associations and co-occurrence within the plastisphere and pondwater communities. Whilst negative correlations were observed between microbial ASVs in the plastisphere and pondwater communities for bacteria and fungi, no negative correlations were observed in the bacterial network for glass or in the eukaryotic networks for glass and pondwater. The average path length (i.e., the number of edges that a path contains) was higher in bacterial pondwater and plastisphere networks to a lesser extent compared to glass, and whilst no difference was observed between the fungal networks, eukaryotic plastisphere networks had a higher average path length than glass or pondwater (Figure S6C). Average degree (i.e. the number of connections to other nodes in the network) was highest in bacterial and fungal plastisphere communities, as opposed to eukaryotic pondwater communities. Overall, significant differences between microbial network associations due to substrate were observed based on Spearman’s coefficient correlation distance matrices (PERMANOVA;* p* ≤ 0.002); networks on glass and plastic significantly differed from those in pondwater (pairwise PERMANOVA; glass vs plastic *P* = 0.632, 0.730 and 0.232 for bacteria, fungi, and eukaryotes, respectively; for eukaryotes, glass vs pondwater *P* = 0.021; all other comparisons *P* ≤ 0.002).

Relative abundances of bacterial genera previously reported as plastic degraders were significantly correlated within glass, plastic and pondwater communities (Fig. 7A, B and C). Potential pathogens identified as significant within the networks were always genera previously reported as putative plastic degraders. These microbes were predominantly in pondwater networks, with 53 out of 235 bacterial ASVs identified. Within the microbial network in the plastisphere and on glass, only *Flavobacterium* sp. and a low abundance of *Rhodoferax* sp. were found as potential plastic degraders, respectively. *Cladosporium* sp. was the only fungal taxon identified as a potential pathogen and plastic-degrader within the fungal networks (Fig. 7E). This suggests strong associations between *Cladosporium* and the plastisphere in wastewater treatment plants. No genera associated with plastic-degradation or pathogens were identified within the eukaryotic communities. Overall, more bacteria presumptively assessed as being plastic-degrading microbes and pathogens were significantly associated within pondwater networks; those microbes were more likely to be found within the plastisphere than glass-associated biofilms. Fewer ASVs were determined to be significantly associated with fungal communities on glass and pondwater compared to the plastisphere.

## Discussion

Many studies have documented the colonisation of microbial communities on plastics in the environment [19, 28, 57, 59, 96]; however, limited studies have investigated bacterial, fungal, and eukaryotic communities on plastics after tertiary treatment in wastewater treatment plants. To address this knowledge gap, we investigated the temporal dynamics of bacterial, fungal, and eukaryotic communities on five plastic types, both artificially UV-aged and non-aged, deployed into the final polishing pond of a wastewater treatment plant in Ōtautahi-Christchurch, Aotearoa-New Zealand. Understanding how the diverse taxa spanning multiple domains of the tree of life associate with plastics in a WWTP system is crucial when identifying and characterising novel potential plastic degraders. Further assessment to determine risks from releasing rafting pathogenic microbes into receiving waterways is important for future mitigation, prevention, and environmental bioremediation initiatives.

We found distinct differences between free-living microbial communities in the ambient WWTP pondwater and those microbes forming biofilm communities on glass and plastics, as previously found by Hong et al. [46] and Oberbeckmann et al. [97]. Immediately following immersion, organic matter quickly accumulates on surfaces in aquatic environments, meaning bioavailable carbon is often present on and within the surface of solid substrates, aiding colonisation by microbes [42, 76]. Attachment for colonisation and subsequent biofilm development can be influenced by physiochemical properties such as surface hydrophobicity, free energy, charge, topography, and roughness [10, 127, 138]. Initial surface adhesion has been previously shown to impact pioneer species recruitment and subsequent community composition; UV-ageing increases the surface roughness and decreases the hydrophobicity of plastic surfaces [111]. A parallel study performed on the same plastic paddles investigated the effects of artificial UV-ageing. Whilst surface cracking and increased crystallinity were observed, there was no indication of biological surface degradation of the plastics in the wastewater treatment plant [128]. In this study, we found no significant variation in beta diversity between communities forming on the artificially weathered plastics compared to non-aged plastics, suggesting that any changes to the surface properties and hydrophobicity via UV-ageing of the plastic pre-deployment did not impact the colonising microbial communities. Similarly, whilst Binda et al. [11] observed decreased hydrophobicity of PE after ten days of direct (i.e., in the air) and indirect (i.e. in solution) UV exposure, there was similar coverage of stable biofilms on UV-aged and non-aged PE after 30 days. Likewise, communities forming on plastic substrates did not significantly differ from those on inert controls, such as highly hydrophilic glass surfaces, as previously observed in other aquatic environments [58, 134]. Tagg et al. [122] demonstrated no clear differences between communities on plastics and silicate substrates. Similar results were found by Parrish & Fahrenfeld [100] in wastewater mesocosms, with microbial communities on polystyrene microplastics resembling those on glass more closely than those on polyethylene microplastics. Relatively strong surface hydrophobicity was determined for non-aged LLDPE, OXO and PA used in our study, with non-aged PET and PLA being more hydrophilic. UV-aged PA was the only artificially aged plastic to become significantly more hydrophilic [128].

Our results suggest several other important factors may drive the composition of microbial communities on substrates. Environmental factors such as light, temperature and biofilm age influence the composition of aquatic biofilms [68, 98, 134]. Similarly, we determined that microbial communities varied over time independent of substrate type, suggesting that these communities may be influenced by resource availability and competition, with early colonisation and succession playing an important role in their assembly [29, 152]. Moreover, Sun et al. [119] demonstrated that marine plastisphere compositions changed over a large depth gradient. Likewise, we found that small variations in depth influenced microbial community compositions. In a parallel study with these samples, Maday et al. [74] found significant variation in the broad-scale functional potentials of WWTP plastisphere communities during the earlier stages of biofilm formation,therefore taxonomic differences we observed at each depth may drive variation in metabolic activity. As the biofilms mature, and light intensity decreases at shallower depths, microbial communities may shift from having more photoautotrophs, such as Cyanobacteria, close to the surface of the water, to more heterotrophs, such as Planctomycetota and Chloroflexi [106, 136], lower in the water column.

Seasonality has previously been shown to influence the composition of plastisphere microbial communities, with differences observed between summer and winter due to abiotic factors such as salinity and water temperatures [114]. We observed an increase in photoautotrophs such as Cyanobacteria at 26 weeks alongside Archaeplastida at 52 weeks, coinciding with the beginning and middle of summer. Cyanobacteria are known to play a vital role within wastewater treatment plants, such as in heavy metal bioremediation, and can increase the oxygen content of pondwater [3]. The nitrogen-fixing abilities of these bacteria have been shown to interact with and support the growth of biodegrading microbial communities associated with petroleum compounds [2] and plastic [73, 125]. However, taxa such as Cyanobacteria, can cause harmful ‘algal’ blooms (HAB) via cyanotoxin production, and they can readily colonise plastics in the marine environment [98, 101]. Archaeplastida encompasses several photosynthetic organisms including green algae and several groups of red algae. Notably, the lack of this phylum on PET and PLA at six months, whilst high abundances were observed on all other substrates and in pondwater, raises questions regarding the influence of polyesters on Archaeplastida. While we did not investigate the pH of the polishing pond whilst sampling, an increased abundance of algae often leads to an elevated pH through the biological absorption of carbon dioxide. Fluctuations in pH can impact the metabolism of microbes present, and in turn, influence not only the microbes present, but their biogeochemical nutrient cycling abilities as well [41, 124].

Overall differences between assemblages on each plastic type were limited; the most abundant taxa at each timepoint were shared across plastic types, as observed in previous studies [116], with greater diversity at earlier biofilm ages and rare genera driving the diversity differences between biofilms. As such, microbes appear to colonise all surfaces with little specificity, providing no clear evidence of plastic-specific communities within the final polishing pond of a wastewater treatment plant. However, the collection of more sample data in future studies (i.e. five replicates instead of three) would reduce the likelihood of generating false negative statistical findings. Additionally, microbial communities on microplastics found in the WWTP system may not be fully represented by the use of plastic paddles due to disparities in their size and fixed location. Vaksmaa et al. [130] determined that subsurface plastic particles hosted more specialised microbes than those on the surface. As larger surfaces are able to host biofilms that are more resistant to external stressors such as shear forces and chemical treatment [111], further investigations are needed to confirm the reliability of using larger plastics as a proxy for microplastic pollution.

Taxa associated with bioremediation of organic matter are often found in WWTP systems and have previously been linked with denitrification, activated sludge stabilisation, and organic decomposition [26, 80, 112, 149]. Previously reported plastic-degrading genera were identified on glass, plastics, and in pondwater, with the most abundant species present in pondwater overall. *Duganella*, *Janthinobacterium*, *Klebsiella, Shewanella* and *Strenotrophomonas* spp. were present exclusively in pondwater, suggesting a preference for living freely within the water column. Within the plastisphere, more plastic biodegrading taxa were abundant at two weeks than at any other time. Notably, thin biofilms were present at this time, meaning higher proportions of the bacteria present within the biofilm may have access to the plastic surface. *Acinetobacter* and *Flavobacterium* spp. were previously reported to degrade 11 plastics (LDPE, PCL, PE, PES, PET, PHA, PHA, PLA, PS, PTS, and PU; [47, 48, 51, 60, 87, 88, 95, 104, 120, 121]) and one plastic (PE; [62]), respectively, and were abundant on glass and plastic, but more so in pondwater in this study. Interestingly, Hong et al. [46] also identified *Flavobacterium* and *Polynucleobacter* as abundant in tertiary treatment pondwater, with the latter suggested originating from activated sludge. Their study observed *Pseudomonas* on polystyrene in the primary stage of treatment, but with a higher abundance in the water. Kelly et al. [55] similarly found high concentrations of *Pseudomonas* on plastics within wastewater effluent. We also found pondwater to have the highest relative abundance of *Pseudomonas*, with the second highest abundance found in biofilms on plastic at two weeks. Potential plastic degraders found exclusively on plastic were observed in low abundance. *Rhodoferax* spp., found in all early biofilms at two weeks (except OXO) and exclusively on LLDPE and PLA after 52 weeks, were previously isolated from soils and reported to biodegrade PHA [120]. Similarly, *Rheinheimera* spp. present only on PA and PLA at two weeks, have been isolated from the deep sea and have been reported to degrade poly-3-hydroxybutyrate-co-3-hydroxyhexanoate films [54], as were *Undibacterium* spp., which were found in all of our plastic biofilms at two weeks, except OXO [90]. Found to be abundant on PA and PET after two weeks and detected at 26 and 52 weeks on LLDPE and PET, respectively, *Leptothrix* spp. have been reported to biodegrade five plastics: poly(butylene adipate-co-terephthalate), poly(butylene succinate-co-terephthalate), PBSA, polycaprolactone, and polyethersulfone (PBAT, PBST55, PCL and PES; [92, 93]).

Assress et al. [7] found fungal genera associated with degrading or transforming organic contaminants such as pesticides, thiocyanate, polyaromatic hydrocarbons and toluene [65, 81, 94], such as *Trichoderma, Acremonium, Talaromyces, Paecilomyces, Cladophialophora* and *Saccharomyces* spp., to be present in WWTPs. Fungal genera with the potential for plastic bioremediation, such as *Alternaria*, *Fusarium*, *Candida*, *Cladosporium*, and *Stemphylium* spp., were detected in our samples at low abundances. The highest abundance of reported plastic biodegrading fungi was observed in mature biofilms, suggesting colonisation and growth as opposed to primary succession. Whilst 35 fungal genera were reported as plastic degraders, *Cladosporium* spp. were the only reported fungal plastic degraders significantly associated with other taxa in the plastisphere co-occurrence network. Previously reported to biodegrade PU, PE, PET, PHB, PBSA and PLA [4, 13, 14, 16, 24, 51, 63, 79, 144], *Cladosporium* spp., were detected on LLDPE, OXO, PA, PET, PLA as well as on glass and in pondwater in low abundance, suggesting plastic-specific colonisation and subsequent plastic biodegradation is unlikely to be occurring. Identification of previously reported plastic degraders does not confirm plastic biodegradation. Putative plastic biodegradation-encoding genes have been identified from our study site in low abundance using metagenomics [74], with the highest abundance found in pondwater. Indeed, the mechanical properties of the plastics used within our study were also tested before and alongside sampling for the deployment. Theobald et al. [128] observed no evidence of major fragmentation, surface alterations or biological degradation throughout the deployment. Therefore, biofilm communities within the final polishing pond of the WWTP are unlikely to biodegrade plastics efficiently and, therefore, are not reliant upon carbon from the plastic polymer for growth.

Tertiary treatment, such as UV radiation, is one of the most important steps to remove microbial pathogens from WWTP systems, allowing treated wastewater to be safely released into the environment. Human bacterial pathogens in our study, including *Aeromonas*, *Klebsiella*, *Legionella*, and *Pseudomonas* spp., are responsible for gastrointestinal, wound, and respiratory infections [132]. Of these, *Pseudomonas* spp., which we found in biofilms but at higher relative abundances in pondwater, are reported as plastic degraders but include opportunistic pathogens such as *Pseudomonas aeruginosa* [107]. *Klebsiella* and *Mycobacterium* spp. were found exclusively in pondwater, suggesting a preference for a planktonic lifestyle. Both are commonly found in wastewater effluent. *Klebsiella* contains a multidrug-resistant opportunistic species, *Klebsiella pneumoniae* [109]. *Mycobacterium* is known as foaming bacteria in activated sludge, but this genus also encompasses numerous pathogens that pose severe risks to mammals, such as *Mycobacterium tuberculosis* and *Mycobacterium leprae* [20, 45, 118]. Opportunistic human pathogens belonging to *Acinetobacter* were observed on all substrates. This genus has been shown to preferentially attach to polystyrene before wastewater treatment, suggesting that plastics may offer protection as a raft may enhance microbial survival during treatment [46, 55, 115]. Similarly, *Legionella* spp. [17, 39] were observed in our study on PET, PLA and pondwater, highlighting the risk of their transportation into the marine environment. Interestingly, our study found high abundances of Bdellovibrionota at the shallowest depth. Often found within activated sludge [150], this bacterial phylum encompasses obligate predatory bacteria capable of preying upon and consuming Gram-negative bacteria in the marine environment, including pathogens such as *E. coli* [71]*. Acremonium* spp., previously reported as frequently found in activated sludge, was present predominantly in biofilms. Most species of this genus are saprophytic, yet some are pathogenic [37].

Alongside the identification of bacterial pathogens, potential fungal pathogens were also investigated. *Nakaseomyces*, a genus comprised of three environmental and three pathogenic yeast species previously assigned to *Candida* [64, 123], were found in low abundances exclusively on plastics at 52 weeks in our study. This genus encompasses two species recently discovered to be human pathogens, *C. nivariensis* and *C. bracarensis*, along with *C. glabrata*, an opportunistic fungal pathogen responsible for up to 29% of *Candida* bloodstream infections [23, 86]. Whilst some *Candida* spp. within our samples were identified as yeasts used for biocontrol, such as *C. saitoana* and *C. oregonensis* [33], other species identified have been reported as emerging pathogens, including *C. norvegica*, *C. sake* and *C. parapsilosis* [53, 129, 147]. The potential pathogens found exclusively on plastics (*Aspergillus*, *Cryptococcus*, *Mucor* and *Talaromyces*), suggest plastic-specific settlement. However, these were only observed in very low relative abundances. Non-viable pathogenic microbes may impact the ability to reliably detect pathogens in wastewater [133]. Interestingly, a metagenomic study conducted within the same WWTP also found these potential fungal pathogens, observing a higher abundance of *Cryptococcus*, *Fusarium* and *Aspergillus* in effluent and pondwater samples compared to influent samples [6]. Our results show variation in the abundance of pathogens over different time points and substrates. A greater relative abundance at 52 weeks implies persistence and replication within a more mature biofilm. The zoonotic protozoan genus *Cryptosporidium* was also identified in our study in low abundance within mature microbial communities on PA. This pathogen is associated with food and waterborne illness [105, 137] and has been reported to adhere to polyethylene microbeads and polyester microfibres in the marine environment [148]. The presence of low concentrations of this parasite poses a threat to both human health and marine life. The presence of these genera within the plastisphere highlights a potential danger of plastic existing and persisting within our waterways. The plastisphere may increase the abundance and survival of these pathogenic microbes, not only in a wastewater treatment plant, but also in the marine environment where effluent from this study is released. However, assessing the viability and pathogenicity of these microbes is essential in determining the actual risks.

Network metrics (total nodes, links, average path length and average degree) revealed plastisphere and bacterial pondwater communities to be more complex than glass-associated communities, with increased positive correlations. Plastisphere networks comprised more nodes, links, and average degree. More ASVs were present, meaning increased diversity and, therefore, more metabolic potential within the community, forming a more stable and complex biofilm. Whilst a longer average path length may suggest reduced efficiency and speed with which metabolites or resources can be passed throughout the biofilm, Santolini & Barabási [113] have previously shown that complex networks are more resilient to environmental changes than simple networks. Pollet et al. [103] observed two-thirds of interactions within bacterial plastisphere networks to be positive and, therefore, concluded that microbial communities may form metabolic functional guilds and behave co-operatively instead of competing. Increased associations within the plastisphere may allow more utilisation of resources and nutrient cycling, which has been shown previously to significantly impact carbon, nitrogen and phosphorus cycles within aquatic systems [21, 36, 146], and may accelerate the release of harmful pollutants, influencing the systems overall health. Interactions within plastisphere communities may also facilitate mutualistic relationships for bioremediation. Joshi et al. [52] revealed increased degradation of LLDPE by a consortium of bacteria interacting cooperatively to degrade polymers more efficiently and effectively than an individual isolated microbe. It is therefore possible that these complex networks formed within plastisphere communities may support co-metabolism as a means of survival. This increases the resilience of plastisphere communities to major environmental perturbations as more microbes may be able to perform the same ecological roles, suggesting the formation of a more stable biofilm. As a result, there is potential for increased pathogenic interactions, with richer communities able to protect pathogens and promote plasmid stability to enhance the exchange of genes associated with pathogenicity or antibiotic resistance. Therefore, the high complexity of microbial communities on plastics might facilitate the degradation of plastics but simultaneously support the exchange of resistance traits and exacerbate the spread of pathogenic microbes.

## Conclusion

With the increasing accumulation of environmental plastics, research into the impacts of this anthropogenic debris on our freshwater and marine ecosystems is critical. Increased effluent disposal on land may cause terrestrial ecosystems to be increasingly exposed to similar risks. Microbial communities associated with plastic and glass were distinct from those in ambient pondwater, with biofilm age and depth influencing community composition and diversity. While microbial communities were not found to be specific to plastic types or conditions (i.e. artificially aged vs pristine plastic), we identified the presence of microbes with the potential to degrade plastic polymers, primarily in pondwater, immature biofilms at two weeks for bacteria and in mature generalist biofilms at 52 weeks for fungi. Likewise, we identified potential pathogenic microbes with the same trends. Increased associations between potential plastic degraders, pathogens and the plastisphere microbial network provide insight into the stability of plastisphere communities. This may have consequences for plastic bioremediation, but also risks associated with plastics persisting in the environment for prolonged periods. The results of this study, alongside observations from previous studies, highlight the importance of investigating all microbes on plastics and in the pondwater of WWTPs and their implications for public health and the environment.

## Supplementary Information

Supplementary Material 1

## Data Availability

Raw sequence reads are available from the NCBI Sequence Read Archive (SRA) under the BioProject ID PRJNA1161030.
