# Supplementary material for: Microbes with plastic-degrading and pathogenic potentials are present on plastics in the final polishing pond of a wastewater treatment plant
Source: Environ Microbiome. 2025 Jul 1;20:80. doi: 10.1186/s40793-025-00737-y (PMC12211008; doi:10.1186/s40793-025-00737-y)
Supplement: Supplementary file 1 — Supplementary Material 1 [file 40793_2025_737_MOESM1_ESM.docx]

**Supplementary Material**

| Substrate | Referred to in this paper as | Base polymer^1,2^ | Known additives | Additive role |
| --- | --- | --- | --- | --- |
| Glass | Glass | Low iron soda-lime float  glass | None | None |
| Linear low-density polyethylene | LLDPE | Innoplus LL7410D1 | 0.25% Irganox B215 (33% Irganox® 1010 (CAS 6683-19-8) and 67% Irgafos® 168 (CAS 31570-04-4)^3^ | Processing and long-term thermal stabilizer |
| Oxo-linear low-density polyethylene | OXO | Innoplus LL7410D1 | 0.2% manganese stearate (CAS3353-05-7) | Oxo-degradable additive |
| Nylon-6 | PA | Ultramid B3S | Talc | Bulking/filler agent |
|  |  |  | 0.5% nylostab S-EED (CAS 422774-15-2) | Stabiliser |
| Polyethylene terephthalate | PET | PAPETCOOL IV0.80 (standard bottle grade) | 0.3% Tinuvin 234 (CAS 70321-86-7) | UV absorbent |
| Polylactic acid | PLA | Ingeo 3052D | < 0.2% ethylene bis(stearamide) (CAS 110-30-5) | Lubricant additive |

**Table S1 | Constitution of substrates used in this study, as previously reported by Laroche *et al.* (2023).** Known additives were added by Scion, Rotorua, NZ.

^1^Inorganic content of PA, PET and PLA polymers was quantified as < 0.1% of the original mass of the samples (below the detection limit) by thermogravimetric testing.

^2^Inorganic content of LLDPE base polymers was quantified as < 0.5% of the original mass of the samples (just above the detection limit) by thermogravimetric testing.

^3^0.13 wt% AO-1076, an antioxidant stabiliser, was detected in virgin polyethylene resin (Bridson *et al.*, 2023).

**Table S2 | PCR primers and conditions for targeting the hypervariable V4 region of the prokaryotic small-subunit ribosomal RNA (16S rRNA) gene, the fungal internal transcribed spacer 2 (ITS2) region of the nuclear ribosomal gene and the hypervariable V4 region of the eukaryotic small-subunit ribosomal RNA (18S) gene.** Underlined sequences represent the Illumina Nextera adaptor overhang required for sample indexing (Kozich *et al.*, 2013).

| Targeted taxa | Gene fragment amplified^1^ | Primer name | Sequence (5’ - 3’) | Annealing temp (°C) | Primer reference |
| --- | --- | --- | --- | --- | --- |
| **Prokaryotes** | 16S rRNA  (V4 region) | 515F_adpt | TCG TCG GCA GCG TCA GAT GTG TAT AAG AGA CAG GTG YCA GCM GCC GCG GTA A | 50^2^ | Parada *et al.* (2016) |
|  |  | 806R_adpt | GTC TCG TGG GCT CGG AGA TGT GTA TAA GAG ACA GGG ACT ACN VGG GTW TCT AAT |  | Apprill *et al.* (2015) |
| **Fungi** | ITS  (ITS2 region) | fITS7_adpt | TCG TCG GCA GCG TCA GAT GTG TAT AAG AGA CAG GTG ART CAT CGA ATC TTT G | 52^3^ | Ihrmark *et al.* (2012) |
|  |  | ITS4_adpt | GTC TCG TGG GCT CGG AGA TGT GTA TAA GAG ACA GTC CTC CGC TTA TTG ATA TGC |  |  |
| **Eukaryotes** | 18S rRNA (V4 region) | Uni18SF_adpt | TCG TCG GCA GCG TCA GAT GTG TAT AAG AGA CAG AGG GCA AKY CTG GTG CCA GC | 54^4^ | Zhan *et al.* (2013) |
|  |  | Uni18SR_adpt | GTC TCG TGG GCT CGG AGA TGT GTA TAA GAG ACA GGR CGG TAT CTR ATC GYC TT |  |  |

^1^Post-PCR purifications were performed using a DNA clean and concentrator-5 kit (Zymo Research, Irvine, CA, USA). All amplicons were sequenced on an Illumina MiSeq instrument by Auckland Genomics Facility (The University of Auckland, New Zealand).

^2^Initial denaturation (94 °C for 3 min); 30 cycles of denaturation (94°C for 45 sec), annealing (50°C for 1 min), extension (72°C for 90 sec); final extension (72°C for 10 min).

^3^Initial denaturation (94 °C for 5 min); 30 cycles of denaturation (94°C for 30 sec), annealing (52°C for 30 sec), extension (72°C for 45 sec); final extension (72°C for 10 min).

^4^Initial denaturation (94 °C for 5 min); 35 cycles of denaturation (94°C for 30 sec), annealing (54°C for 30 sec), extension (72°C for 45 sec); final extension (72°C for 7 min).

**Figure S1 | Relative abundance (%) of genera identified in (A) bacterial, (B) fungal and (C) eukaryotic mock microbial community controls.** Mock extraction data from fungi and eukaryotes were omitted as no reads were retained after DADA2 processing.

**Figure S2 | Rarefaction curves of (A) prokaryotic, (C) fungal and (E) eukaryotic communities, rarefied to 1000, 2945, and 250 DNA sequence reads, respectively.** Chao 1, Shannon and inverse Simpson indices of rarefied (B) prokaryotic, (D) fungal and (F) eukaryotic communities on plastics, glass and in pondwater from the final polishing pond of the wastewater treatment plant in Ōtautahi-Christchurch, Aotearoa-New Zealand. The box shows the 25^th^ and 75^th^ percentile, the line inside each box represents the median value and the dots represent data outliers. Error bars represent standard error, and lowercase letters for each column represent statistical significance among substrates at each age (Tukey's HSD *P* < 0.05).

**Figure S3 | Non-metric multidimensional scaling (NMDS) ordination for the visualisation of Bray-Curtis similarities of (A) prokaryotic, (B) fungal, and (C) eukaryotic communities on substrates in the final polishing pond of the Christchurch Wastewater Treatment Plant in Ōtautahi-Christchurch, Aotearoa-New Zealand.** Each point represents one sample, labelled according to substrate type (colour) and biofilm age (shape). Closer proximity of points indicates increasing compositional similarity among those data.

**Figure S4 | Relative abundances (%) of (A) all prokaryotic phyla and (B) Cyanobacteria within communities from the final polishing pond of the Christchurch Wastewater Treatment Plant in Ōtautahi-Christchurch, Aotearoa-New Zealand (A-NZ).** Each bar represents the stacked relative abundance, labelled based on depth (e.g. Glass 1 is the top level, Glass 2 is the middle level and Glass 3 is the bottom level), collected at four different times. Each colour corresponds to a particular phylum.

**Figure S5 | Relative abundances (%) of (A) fungal and (B) eukaryotic phyla within communities from the final polishing pond of a wastewater Treatment Plant in Ōtautahi-Christchurch, Aotearoa-New Zealand (A-NZ).** Each bar represents the stacked relative abundance, labelled based on depth (e.g. Glass 1 is the top slide, Glass 2 is the middle slide and Glass 3 is the bottom slide), collected at four different times. Each colour corresponds to a particular phylum.

**Figure S6 | Bar charts summarising the (A) total number of nodes, (B) total links (stacked), (C) average path length and (D) average degree associated with each bacterial, fungal, and eukaryotic network based on substrate.** Pattern fill for B) total links represents the Spearman’s correlation of each link (i.e. a striped pattern shows the total number of negative correlations, and a solid pattern shows the total number of positive correlations). Visualised network interactions are shown in Figure 7.
